# DUT‐58 (Co) Derived Synthesis of Co Clusters as Efficient Oxygen Reduction Electrocatalyst for Zinc–Air Battery

**DOI:** 10.1002/gch2.201700086

**Published:** 2017-11-29

**Authors:** Lichao Gao, Shuai Chen, Rongsheng Cai, Quansheng Zhao, Xiaoliang Zhao, Dongjiang Yang

**Affiliations:** ^1^ School of Environmental Science and Engineering Collaborative Innovation Center for Marine Biomass Fibers Materials and Textiles of Shandong Province Qingdao University Qingdao 266071 P. R. China; ^2^ State Key Laboratory of Coal Conversion Institute of Coal Chemistry Chinese Academy of Science Taiyuan 030001 P. R. China; ^3^ Nanoscale Physics Research Laboratory School of Physics and Astronomy University of Birmingham Birmingham B15 2TT UK; ^4^ Queensland Micro‐ and Nanotechnology Centre and School of Natural Sciences Griffith University Nathan Brisbane QLD 4111 Australia

**Keywords:** Co nanoclusters, graphene oxide, metal–organic framework, oxygen reduction reaction, zinc–air batteries

## Abstract

To meet the requirement of fuel cells and metal–air batteries, non‐noble metal catalysts have to be developed to replace precious platinum‐based catalysts. Herein, Co nanoclusters (≈2 nm) are anchored on nitrogen‐doped reduced graphene oxide (Co/N‐r‐GO) by using DUT‐58 (Co) metal–organic framework and GO as precursors. Compared with single‐atom catalysts usually with ultralow concentration (<0.5 wt%), Co nanoclusters are more beneficial to break the O—O bond to ensure four electronic way for oxygen reduction reaction (ORR), since they can provide more adsorption centers for reactants. Therefore, as expected, the sample with 6.67 wt% Co content (Co/N‐r‐GO‐5%‐850) exhibits better ORR activity with a higher half‐wave potential of 0.831 V, a more positive onset potential of 0.921 V than Pt/C, and a comparable limiting current density in alkaline medium. The Co nanoclusters enhance the catalytic performance for ORR in three aspects: quantum size effects, metal–support interactions, and low‐coordination environment of metal centers. Furthermore, the sample is assembled into a zinc–air battery as the outstanding durable ORR catalyst. It displays a higher specific capacity (795 mAh g^−1^ at the current density 50 mA cm^−2^) and power density (175 mW cm^−2^) than Pt/C (731 mAh g^−1^ and 164 mW cm^−2^, respectively).

## 1 Introduction

The oxygen reduction reaction (ORR) has been one of the most fundamentally and technologically important electrochemical reactions for fuel cells and zinc–air batteries (ZABs).^1^, ^2^, ^3^, ^4^, ^5^ Until now, Pt based nanomaterials and its alloys have been the most efficient electrocatalyst for ORR.^6^, ^7^ However, their high cost, the scarcity, and poor durability prevent large‐scale practical application of business.^8^, ^9^, ^10^ To overcome these problems, heteroatom S, N‐doped or codoped carbon stuffs and transition metal based carbon materials as the electrocatalysts have been widely studied as effective candidates to replace precious metal catalyst for the ORR in ZABs so far.^4^, ^11^, ^12^ For example, Co‐based materials have been widely researched as one of the most considerable alternative electrocatalysts for the ORR.^13^, ^14^, ^15^, ^16^

Metal–organic frameworks (MOFs) have been widely studied not only due to their potential applications such as in optics and gas storage, but also their catalytic performance as the precursor in ORR.^17^, ^18^, ^19^ Recently, N‐doped reduced graphene oxide (r‐GO) with unique 2D structure and excellent conductivity has been considered as catalytic materials for ORR.^20^ Since the direct carbonation of MOF crystals can breakdown their inside structure and then resulting materials cannot achieve required electronic conductivity for ORR. Thus, anchoring MOF on GO and then carbonizing can enhance the conductivity and meet the requirement.^21^, ^22^, ^23^ Co‐MOFs based on N, S, or other heteroatoms‐containing ligands will generate cobalt based S, N, or other heteroatom doped carbon materials through the pyrolysis. These will provide porous architecture, electric conductivity, and rich catalytic active sites, which all help the catalytic performance for ORR. However, previous studies reported Co nanoparticles (NPs) electrocatalysts derived from MOFs have uniform size distribution ranging from 10 to 20 nm.^24^, ^25^ As we know, the smaller particles show a better catalytic performance for ORR because they can provide more catalytic centers and active sites which promote a faster electron and mass transfer process.^26^ Therefore, we urgently need to prepare new performance superior catalysts with smaller Co NPs sizes.

Recently, single‐atom catalysts as a novel type of catalysts with ultrasmall size and excellent catalytic performance have successfully attracted our attention.^27^, ^28^ Although the single atom structure can be maximized utilized with high activity per unit quality, its own nature is still controversial. First of all, single‐atom active sites are vital to achieve high selectivity for hydrogen peroxide from ORR.^29^ Catalysis processes involve the interaction of reactants, where the conversion of reactants to new chemicals requires the breaking and forming of chemical bonds.^30^ As we all know, traditional NPs catalyst is usually four electronic (O_2_ +  4H^+^ +  4e^−^ → 2H_2_O) ways in the process of ORR, which requires more than two atoms in the adsorption of reactants together.^29^, ^31^ Therefore, it is important that more than two atoms break the oxygen–oxygen bond to ensure 4e^−^ pathway. Second, it remains challenging to develop highly active ORR catalysts based on signal atoms metal materials because of low loading of signal atoms metal catalysts.^29^ All in all, we need find a new way to solve these problems. Metal nanoclusters are made up of several to some tens of atoms and possessed size ≈2 nm, which is beneficial for ORR.^32^, ^33^ Metal nanoclusters are potentially more favorable for ORR, since metal clusters can solve the problem of low loading and single catalyst activity center. Furthermore, metal clusters have a distinct small size and high proportion of low‐coordinated surface atoms, which contribute to the absorption of oxygen atoms to promote ORR.

Herein, we report a new class ORR catalyst, Co clusters (≈2 nm) anchored on nitrogen‐doped reduced graphene oxide (r‐GO) through pyrolysis of N‐rich Co‐MOF (DUT‐58) and GO composite in N_2_ atmosphere. Compared with a noble metal Pt/C catalyst, the sample, Co/N‐r‐GO‐5%‐850 shows more positive half‐wave potential (0.831 V vs 0.821 V of Pt/C), a higher onset potential (0.921 V vs 0.915 V of Pt/C), better long‐term stability, and comparable limiting current density at 0.2 V in alkaline media. And furthermore, Co/N‐r‐GO‐5%‐850 as the outstanding durable ORR catalyst for ZABs displays a higher specific capacity (795 mAh g^−1^) at the current density 50 mA cm^−2^ and power density (175 mW cm^−2^) than Pt/C (731 mAh g^−1^ and 164 mW cm^−2^, respectively).

## 2 Results and Discussion

**Scheme 1:**
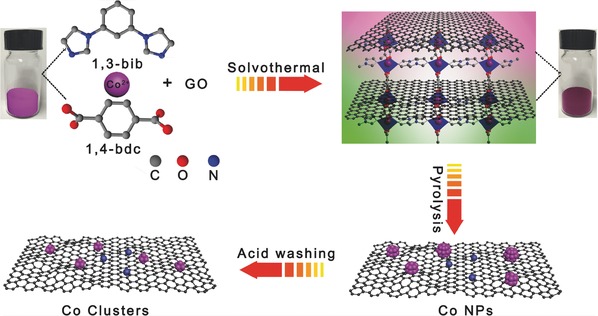
Illustration of the preparation of Co/N‐r‐GO‐850.

The synthesis path to Co/N‐r‐GO‐850 is charted in **Scheme** **1**, a series of catalysts with different GO contents (−2, −5, −10, and −15 wt%), Co/N‐r‐GO (−2, −5, −10, and −15%), were prepared from the mixture of DUT‐58 and GO by a solvothermal and calcination process. DUT‐58 was chosen as the one of the catalyst precursors owing to its porous framework and high nitrogen content from the ligand.^34^ GO as the other one precursor converted into r‐GO through the calcinations which will generate better conductivity.^35^ First, we prepared DUT‐58@GO based on cobalt salt, ligands, and GO through the solvothermal process. Second, the ligand from DUT‐58 and GO were transferred into N‐doped graphitic carbon during the carbonization process in N_2_ atmosphere. Meanwhile, the Co^2+^ ions were reduced to Co NPs. Finally, we obtained Co clusters anchored on nitrogen‐doped r‐GO by acid washing. The details were listed in next experimental section. In these processes, pyrolysis temperature is an important factor for the ORR performance besides the amount of GO. The ORR polarization curves of the catalysts with different carbonization temperature in Figure S1a,b (Supporting Information). It is obvious that the sample shows the best ORR activity at 850 °C. This is probably because 850 °C annealing can make Co/N‐r‐GO‐5%‐850 have an optimal balance of surface area, active site density, and electrical conductivity.^36^

**Figure 1:**
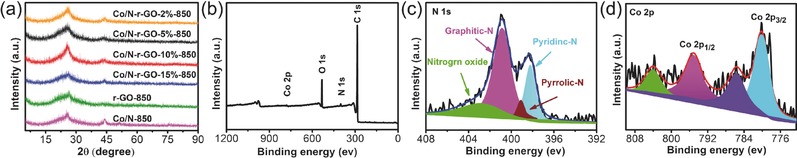
a) XRD patterns of r‐GO‐850, Co/N‐850, Co/N‐r‐GO (−2, −5, −10, and −15%)‐850. The XPS spectra of Co/N‐r‐GO‐5%‐850: b) full spectrum; c) N 1s; d) Co 2p peak.

The powder X‐ray diffraction (XRD) measurements were performed for offering the phase structures of the catalysts. We can find the as‐synthesized DUT‐58 samples are in accord with the simulated one, meaning that the synthesized DUT‐58 is phase‐pure. But no obvious diffraction peak belonged to GO from Figure S2a (Supporting Information), which displays XRD of DUT‐58 and different contents GO composites. In **Figure** **1**a, it displays the XRD patterns of r‐GO‐850, Co/N‐850, and Co/N‐r‐GO (−2, −5, −10, and −15%)‐850. From Figure 1a, every sample reveals a broad peak at 2θ = 26° which is assigned to typical (002) planes of graphitic carbon originated from DUT‐58 and GO through the calcinations.^23^ The very weak peaks appearing at 44.3° are indexed to (111) planes of Co.^24^ But the graphitic peak at 26° became more intense due to the removal of impurities and bulky cobalt after acid washing in Figure S2b (Supporting Information).

X‐ray photoelectron spectroscopy (XPS) of Co/N‐r‐GO‐5%‐850 is conducted to evaluate its detailed elemental composition and chemical valence. As depicted in Figure 1b, there are C, N, O, and Co elements in the sample Co/N‐r‐GO‐5%‐850. The N actually derived from the decomposed 1,3‐bis(imidazole‐1‐yl) benzene (1,3‐bib) ligand, which also provides an evidence for the successful doping. The spectrum of N 1s can be deconvoluted to four peaks at 398.2, 399.2, 400.8, and 402.7eV, corresponding to Pyridinic‐N, Pyrrole‐N, Graphitic‐N, and N‐oxide, respectively (Figure 1c).^37^, ^38^, ^39^ According to the previous reports, all these types of N except the N—O can enhance the ORR performance, especially the Pyridine‐N.^37^ Furthermore, the peaks at 780.1 and 795.2 eV display the binding energies of the Co 2p_3/2_ and Co 2p_1/2_, respectively. And their corresponding satellite peaks are at 785.3 and 803.9 eV, respectively (Figure 1d).^38^ The O 1s spectrum shows two contributions, the presence of O element peaks at 531.6 and 533.1 eV, which can be attributed to the C—O^39^ species and O^40^ from surface H_2_O centered, respectively (Figure S3, Supporting Information). In the C 1s spectrum (Figure S4, Supporting Information), the peaks at 284.6, 285.7, and 288.5 eV are attributed to graphitic carbon, C—O^24^ and C=O^41^ bonds, respectively.

In the Raman spectra, two characteristic peaks D and G bands are shown at 1353 and 1594 cm^−1^, which are associated with disordered carbon and graphitic carbon, respectively (Figure S5, Supporting Information). The low intensity ratio (*I*_D_/*I*_G_ < 1) is due to highly graphitization support in these samples. In our work, the value of *I*_D_/*I*_G_ of the sample, Co/N‐r‐GO‐5%‐850, with the best catalytic performance is 0.95 as shown in Figure S5 (Supporting Information). In addition, the content Co clusters (6.67 wt%) were measured by thermos gravimetric analysis in the Co/N‐r‐GO‐5%‐850 composite catalyst (Figure S6, Supporting Information). This content is far higher than the reported single atom (Co, Fe, etc.) contents.^42^, ^43^, ^44^

The Brunauer–Emmett–Teller specific surface areas, average pore size, and pore volume of the Co/N‐r‐GO (−2, −5, −10, and −15%)‐850 samples are listed in Table S1 (Supporting Information), which are in the ranges of 165–206 m^2^ g^−1^, 3.8–7.9 nm, 0.20–0.38 cm^3^ g^−1^, respectively. Furthermore, Barrett–Joyner–Halenda pore size distribution and N_2_ adsorption–desorption isotherms of these samples are displayed in Figure S7 (Supporting Information). Apparently, all of the samples show a hysteresis loop owing to the existence of the mesopores in the structure.^45^ It is expected that porous nanostructures are conducive to the O_2_ adsorption and the charge transport in ORR process.^8^, ^46^

**Figure 2:**
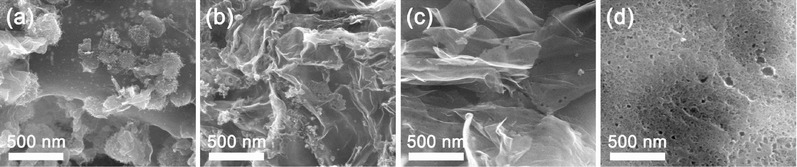
SEM images of a) Co/N‐r‐GO‐2%‐850, b) Co/N‐r‐GO‐5%‐850, c) Co/N‐r‐GO‐10%‐850, d) Co/N‐r‐GO‐15%‐850.

In **Figure** **2**, the morphology and structure of the Co/N‐r‐GO (−2, −5, −10 and −15%)‐850 materials can be observed on field emission scanning electron microscopy images. The samples, the Co/N‐r‐GO (‐2, ‐5, and ‐10%)‐850, exhibit a similar morphology. In Figure 2b,c, r‐GO is well dispersed with thin and wrinkled sheets. With the increase of r‐GO content, the Co/N‐r‐GO‐15%‐850 presented a distinct feature, the porous honeycomb structure due to random aggregation of the constituents (Figure 2d).

**Figure 3:**
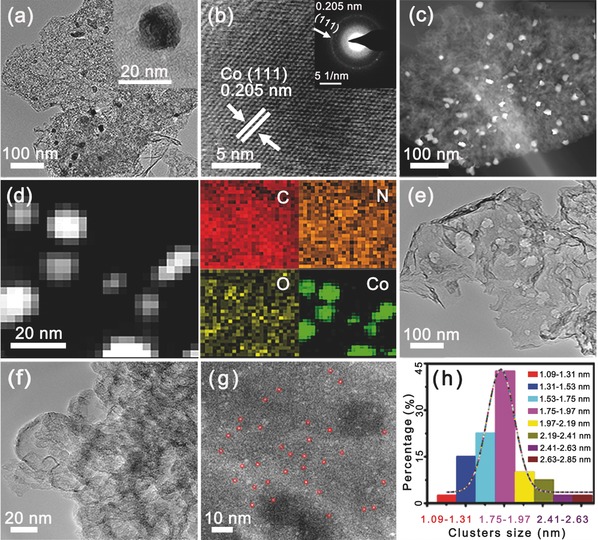
Images of Co/N‐r‐GO‐5%‐850‐NA: a) TEM; Inset: the HRTEM of the particle. b) The HRTEM images and SAED pattern. c) TEM image. d) The HAADF‐STEM and EDS elemental mapping (C, N, O, and Co). e–h) images of Co/N‐r‐GO‐5%‐850: e) TEM image, f) HRTEM image, g) HAADF‐STEM image, h) the size distribution histogram of the clusters.

For comparison, the sample not acid washing named Co/N‐r‐GO‐5%‐850‐NA was prepared. The transmission electron microscopy (TEM) images of Co/N‐r‐GO‐5%‐850‐NA are presented in **Figure** **3**a–c and Figure S8a–c (Supporting Information). TEM of the sample (Figure 3a) reveals that cobalt metal NPs with an approximate diameter of 8–15 nm (inset, Figure 3a) are embedded in the carbon sheet. Furthermore, high‐resolution TEM (HRTEM) images display the fringe spacing of 0.205 nm corresponding to the (111) plane of Co NPs (JCPDS no.97‐062‐2435) (Figure 3b). The selected‐area electron diffraction (SAED) pattern (inset, Figure 3b) is consistent with the (111) plane of HRTEM results. TEM image in Figure 3c reveals that cobalt nanoparticles are anchored uniformly. As presented in Figure S8c (Supporting Information), the Co NPs are encapsulated by the graphite carbon which originates from not only GO but also the C‐containing ligands of DUT‐58. Figure 3d displays the representative C, N, O, and Co mappings of Co/N‐r‐GO‐5%‐850‐NA by high‐angle annular dark‐field scanning transmission electron microscope (HAADF‐STEM) with energy‐dispersive X‐ray spectrometry (EDS) mapping. N‐mapping demonstrates that N atoms are homogenously distributed over the entire structure, deriving from the decomposition of N‐containing ligands of DUT‐58. Similarly, C atoms result from the C‐containing linkers and GO. It is noticeable that Co NPs with a uniform distribution from EDS mapping are coincided with the ones observed from HAADF‐STEM. The TEM images of Co/N‐r‐GO‐5%‐850 are shown in Figure 3e–g and Figure S8d–f (Supporting Information). In Figure 3e, we cannot find Co clusters in TEM images, probably due to the fact that the most of cobalt metals decrease the size sharply or dissolved by acid washing. Hence, we can only see clearly the pure carbon materials in Figure 3e,f and Figure S8d (Supporting Information). To further depict the structure of Co/N‐r‐GO‐5%‐850, HAADF‐STEM images are shown in Figure 3g and Figure S8e,f (Supporting Information). It is clearly shown that Co clusters with main diameter sizes of ≈1–2 nm are uniformly distributed on N‐doping carbon materials. The size distribution of Co clusters is estimated from HAADF‐STEM image in Figure 3h, mainly centered between 1.3 and 2.4 nm. This result is basically consistent with Figure 3g.

**Figure 4:**
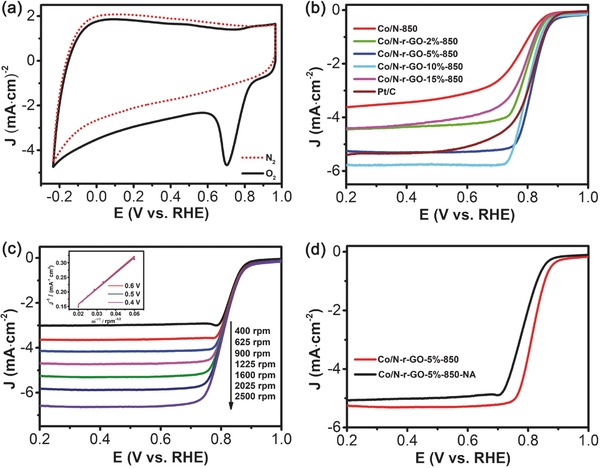
a) Co/N‐r‐GO‐5%‐850 CV curves in O_2_‐saturated (black line) and N_2_‐saturated (red line) 0.1 M KOH aqueous solution. b) LSV curve of Co/N‐850 and Co/N‐r‐GO (−2, −5, −10, and −15%)‐850 comparison with Pt/C in O_2_‐saturated 0.1 M KOH at 1600 rpm. c) LSV curves of Co/N‐r‐GO‐5%‐850 at various rotation speeds; inset: the K–L plots from LSV curves at 0.6, 0.5, 0.4 V. d) LSV curves of Co/N‐r‐GO‐5%‐850‐NA and Co/N‐r‐GO‐5%‐850 at 1600 rpm.

In order to evaluate the ORR activity of Co/N‐r‐GO‐5%‐850, the cyclic voltammetry (CV) curve in **Figure** **4**a shows an apparent oxygen reduction peak for the sample in the O_2_‐saturated 0.1 M KOH aqueous solution, but disappears in the N_2_‐saturated 0.1 M KOH aqueous solution at a scan rate of 50 mV s^−1^, which indicates no reaction happened in the N_2_‐saturated solution. The obvious oxygen reduction peak presents to ≈0.71 V vs reversible hydrogen electrode (RHE) in the O_2_‐saturated solution for the Co/N‐r‐GO‐5%‐850, demonstrating that it can remarkably catalyze the ORR. The electrochemical catalytic activity of Co/N‐850, Co/N‐r‐GO (−2, −5, −10, and −15%)‐850 and 20 wt% Pt/C were recorded by linear sweep voltammetry (LSV) with a rotating disk electrode (RDE) at scan rate 5 mV s^−1^ in O_2_‐saturated 0.1 M KOH at 1600 rpm (Figure 4b). As shown in Figure 4b and Figure S9 (Supporting Information), the Co/N‐r‐GO‐5%‐850 shows a half‐wave potential at ≈0.831 V (vs RHE) and onset potential at 0.921 V, which is more positive than 20 wt% Pt/C catalyst (0.821 V vs RHE and 0.915 V, respectively) and other samples. Our onset potential was slightly superior to the reported materials (0.92 V vs 0.91 V).^24^, ^47^ Meanwhile, the limiting current density of Co/N‐r‐GO‐5%‐850 is −5.3 mA cm^−2^, which is highly comparable to that of Pt/C (−5.4 mA cm^−2^). It indicates that proper amounts of r‐GO (5 wt%), Co clusters, and mesoporous carbon systems are conducive to the enhancement of the ORR performances. From Figure 4c, the current density of Co/N‐r‐GO‐5%‐850 gradually increased with the rise of RDE rotation speed from 400 to 2500 rpm owing to the decrease of interface concentration polarization between electrolyte and electrode. The related K–L plots reveal the inverse current density (*j* ^−1^) as a function of opposite of square root of rotating speed (ω^−1/2^) at different potential values (inset, Figure 4c). It represents a good linearity at different rotation speeds, and the *n* value is about 3.75 in the voltage range of 0.4–0.6 V, indicating the ORR process catalyzed by Co/N‐r‐GO‐5%‐850 is the four‐electron transfer pathway.

To verify the deeper effect of acid washing (Figure S1, Supporting Information), through the comparison test, the sample of Co/N‐r‐GO‐5%‐850 by acid washing with the smaller Co size has a better onset potential, half‐wave potential, and limiting current density (Figure 4d). In addition, the different acid washing times for the sample of Co/N‐r‐GO‐10%‐850 were tested for ORR performance (Figure S9, Supporting Information). On the one hand, the acid washing can remove the surface contaminants with cobalt nitrate and obstruct the debris of channels, so that more holes and activation sites are exposed to improve ORR performance.^4^ On the other hand, with decrease in the size of metal particles, the catalytic performance per metal particle usually enhances from three aspects: quantum size effects, metal–support interactions, and low‐coordination environment of metal centers.^46^ Indeed our results confirm that that the ORR performance of Co/N‐r‐GO‐5%‐850 is better than the samples without acid treatment.

To quantify the ORR pathway, a rotating ring‐disk electrode technique is performed to monitor the formation of HO_2_^−^ during the ORR process in Figure S11 (Supporting Information). The H_2_O_2_ yield of the Co/N‐r‐GO‐5%‐850 catalyst is below 13% at all potentials, and transfer number of 3.75 is keeping with the K–L plot result. In addition, we further compared the ORR activity of commercial Pt/C with Co/N‐r‐GO‐5%‐850 catalyst (Figures S10 and S12, Supporting Information). It is amazing to find that onset potential and half‐wave potential of the Co/N‐r‐GO‐5%‐850 are slightly better than 20 wt% Pt/C. Excellent ORR activity of the Co/N‐r‐GO‐5%‐850 catalyst was also gleaned from the much smaller Tafel slope of 58 mV per decade at low over‐potentials (Figure S13, Supporting Information) than Pt/C (61 mV per decade) in 0.1 M KOH. Additionally, Co clusters with the size of 1–2 nm play a crucial role in improving ORR performance. The higher metal clusters catalyze activity is derived from the high fraction of low‐coordinate surface atoms for oxygen molecules adsorption than the close‐packed counterparts.^48^, ^49^ Compared with metal particles, the utilization of the nanoclusters as electrocatalysts can effectively improve the efficiency of ORR.^50^

The durability of Co/N‐r‐GO‐5%‐850 experiment was performed at a constant potential of 0.56 V vs RHE for 20 000 s in an O_2_‐saturated 0.1 M KOH solution at a rotation rate of 1600 rpm (Figure S14, Supporting Information). It is observed that the samples exhibit a slight decrease (8%) over 20 000 s, indicating that the stability of Co/N‐r‐GO‐5%‐850 is much superior to the commercial Pt/C catalyst (a decrease about 22%) in the alkaline medium electrolyte.

**Figure 5:**
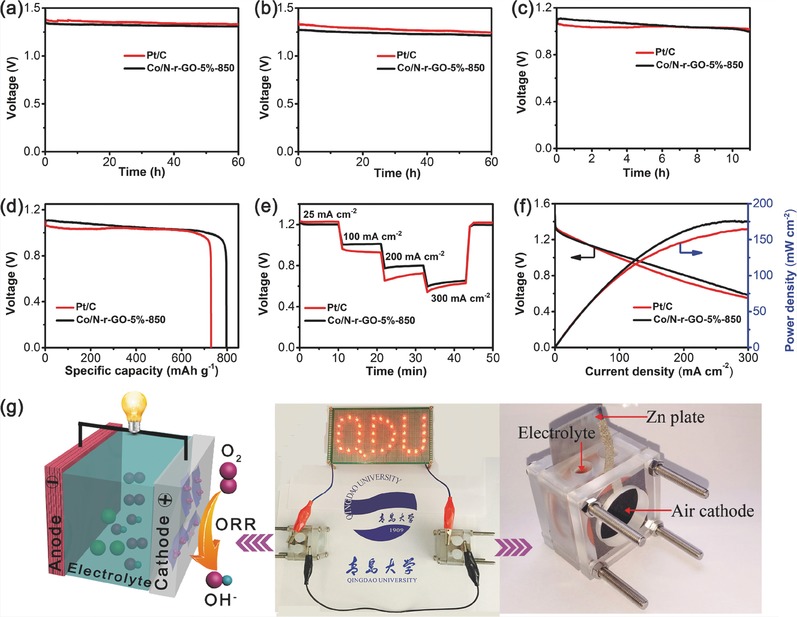
Curves of ZABs driven by Co/N‐r‐GO‐5%‐850 and 20% Pt/C: typical galvanostatic discharge curves at current densities of a) 1 mA cm^−2^, b) 5 mA cm^−2^, c) and 50 mA cm^−2^. d) Specific capacities normalized to the mass of the consumed zinc of ZABs at current density of 50 mA cm^−2^. e) The Zn–air batteries from low current densities to high current densities. f) The polarization and power density curves. g) The schematic diagram of the ZAB and optical image of LEDs lightened by Co/N‐r‐GO‐5%‐850‐based ZABs.

ZABs are regarded as a good candidate to substitute lead–acid batteries which present a serious damage to the environment and human health. In order to demonstrate the ability of practical application of the catalyst, the discharge voltage profiles of the assembled ZABs were consisted of Co/N‐r‐GO‐5%‐850 loading on carbon papers as air cathode (as shown in **Figure** **5**g). The galvanostatic discharge curves depicted in Figure 5a–c clearly reveal that the performance of Co/N‐r‐GO‐5%‐850 based battery is very close to Pt/C at current densities of 1, 5, and 50 mA cm^−2^. Furthermore, the performance with a higher voltage (≈1.08 V) is superior to Pt/C at the current density 50 mA cm^−2^ in Figure 5c. Besides, both potentials of Co/N‐r‐GO‐5%‐850 and Pt/C just decrease slightly after galvanostatically discharged for 60 h at current densities of 1 and 5 mA cm^−2^. Co/N‐r‐GO‐5%‐850 displays an excellent durability on account of the good ORR stability. The specific capacity of the Co/N‐r‐GO‐5%‐850 based on ZAB normalized to the mass of consumed zinc was evaluated to be 795 mAh g^−1^, higher than Pt/C (731 mAh g^−1^) at the discharge density of 50 mA cm^−2^ (Figure 5d). The ZAB catalyzed by Co/N‐r‐GO‐5%‐850 also showed excellent rate capability and a fast dynamic response (Figure 5e). In addition, the polarization and power density curves of Co/N‐r‐GO‐5%‐850 and Pt/C as the cathodes are presented in Figure 5f. The Co/N‐r‐GO‐5%‐850 displays a current density of ≈114 mA cm^−2^ higher than Pt/C (99 mA cm^−2^) at 1.0 V. The peak power densities are 175 and 164 mW cm^−2^ for Co/N‐r‐GO‐5%‐850 and Pt/C in Figure 5f, respectively. In contrast to the literature reported,^51^, ^52^ our specific capacity (795 mAh g^−1^) and power density (175 mW cm^−2^) have a distinct advantage. Simultaneously, we have tested the ZAB performances of Co/N‐r‐GO‐10%‐850 (Figure S15, Supporting Information). As exemplified in Figure 5g, two ZABs in series prepared with Co/N‐r‐GO‐5%‐850 as cathode catalyst were connected to light the light‐emitting diodes (LED) which operate at a minimum voltage of 2.0 V. Meanwhile, a digital photograph of the ZAB is shown in the Figure 5g.

## 3 Conclusion

In conclusion, porous graphitized carbon supported the cobalt clusters, entitled Co/N‐r‐GO were prepared from the mixture of DUT‐58 and GO through a pyrolysis process. The obtained Co/N‐r‐GO‐5%‐850 displays more positive half‐wave potential (0.831 V vs 0.821 V of Pt/C), a higher onset potential (0.921 V vs 0.915 V of Pt/C), better long‐term stability, and comparable limiting current density at 0.2 V in alkaline media. What is more, it also exhibits a better long‐term stability with ignorable voltage reduction for 60 h at current densities 1 and 5 mA cm^−2^, a higher specific capacity (795 mAh g^−1^), peak power density (175 mW cm^−2^), and a current density (≈114 mA cm^−2^) at 1.0 V as an air cathode for ZAB device than Pt/C (731 mAh g^−1^; 164 mW cm^−2^; ≈99 mA cm^−2^, respectively). The excellent catalytic properties of synthesized samples benefit from Co nanoclusters (≈2 nm) which provide more adsorption centers for O_2_ to ensure four electronic ways for ORR and then its ultrasmall size enhances the catalytic performance from three aspects: quantum size effects, metal–support interactions, and low‐coordination environment of metal centers. Anyhow, we have synthesized a new catalyst derived from MOF with a good performance in the galvanostatic discharge ZAB, which acts as the non‐noble metal catalyst that probably replaces the expensive commercial Pt/C in ZAB and alkaline fuel cell.

## Conflict of Interest

The authors declare no conflict of interest.

## Supporting information

Supplementary
