# Supplementary material for: DUT‐58 (Co) Derived Synthesis of Co Clusters as Efficient Oxygen Reduction Electrocatalyst for Zinc–Air Battery
Source: Glob Chall. 2017 Nov 29;2(1):1700086. doi: 10.1002/gch2.201700086 (PMC6607359; doi:10.1002/gch2.201700086)
Supplement: Supplementary file 1 — Supplementary [file GCH2-2-1700086-s001.pdf]

Copyright WILEY-VCH Verlag GmbH & Co. KGaA, 69469 Weinheim, Germany, 2017.

Supporting Information

for *Global Challenges,* DOI: 10.1002/gch2.201700086

DUT-58 (Co) Derived Synthesis of Co Clusters as Efficient Oxygen Reduction Electrocatalyst for Zinc–Air Battery

Lichao Gao, Shuai Chen, Rongsheng Cai, Quansheng Zhao, Xiaoliang Zhao, and Dongjiang Yang**

Supporting Information

DUT-58 (Co) Derived Synthesis of Co Clusters as Efficient Oxygen Reduction Electrocatalyst for Zinc-Air Battery

Lichao Gao, Shuai Chen, Rongsheng Cai, Quansheng Zhao, Xiaoliang Zhao, and Dongjiang Yang**

Experimental Section

Materials: Cobalt nitrate hexahydrate ($Co(NO_3)_2·6H_2O$), N,N-Dimethylformamide (DMF), and ethylalcohol were purchased from Sinopharm Chemical Reagent (Shanghai, China) and used without further purification. P-phthalic acid (1,4-bdc) were purchased from J&K Scientific Ltd. Hydrochloric acid was purchased from the Shuangshuang Chemistry Company (Yantai, China). 1,3-bis(imidazole-1-yl) benzene (1,3-bib) was purchased from Jinan Camolai Trading Company (Jinan, China). Deionized (DI) water (>18 MΩ) was used as a solvent in the synthesis. GO was purchased from Institute of Coal Chemistry, Chinese Academy of Sciences. Nitrogen gases were supplied in cylinders by Heli factory with 99.999% purity.

Synthesis of Co-MOF (DUT-58): The synthesis of Co-MOF (DUT-58) nanocrystals was based on a previous method with making some modifications.[1] Typically, $Co(NO_3)_2·6H_2O$ (105 mg, 0.4 mmol) and 1,4-bdc (66 mg, 0.4 mmol) were dissolved in 18 mL of DMF. 1,3-bib (84 mg, 0.4 mmol) was dissolved in 2 mL of DMF. After ultrasonic for 30 min, the solutions were mixed, and then turned into a Teflon vessel (50 mL). The vessel was heated at 120 °C with a heating rate of 1.2 °C min^{-1} in an oven for 36 h followed by slow cooling to room temperature at 0.4 °C min^{-1}. The purple crystals were collected through filtration and washed with DMF and C_2H_5OH several times and dried under vacuum.

Synthesis of Co-MOF/GO (2, 5, 10 and 15 wt%): $Co(NO_3)_2·6H_2O$ (105 mg, 0.4 mmol) and 1,4-bdc (66.4 mg, 0.4 mmol) were dissolved in 18 mL of DMF. 1,3-bib (84 mg,

0.4 mmol) was dissolved in 2 mL of DMF. Then we mix these two solution, and different content of GO (2, 5, 10, and 15 wt%: based on the total quality of initial solid materials) were added to the mixtures. The vessel was heated at 120 °C with a heating rate of 1.2 °C min^{-1} in an oven for 36 h followed by slow cooling to room temperature at 0.4 °C min^{-1}. The purple crystals were collected through filtration and washed with DMF and C_2H_5OH several times and dried under vacuum.

Synthesis of Co clusters Hybrid (Co/N-r-GO-850): The above prepared Co-MOF/GO hybrid were transferred to tube furnace and heated from room temperature to 850 °C at a heating rate of 2 °C min^{-1} for 2 h under an N_2 atmosphere before cooling down to room temperature. Reduced GO is considered to be a special type of support to increase activity and stability, because its sp^2-hybridized carbon structure which provide better conductivity.[2-3]

To eliminate Co/N-r-GO hybrid catalyst surface impurities and bulky cobalt, the sample were stirred at room temperature in 3.0 M HCl solution for 12 hours, and afterward washed to neutral with deionization water. The synthesis path to Co/N-r-GO-850 is charted in Scheme 1.

Characterization: The phase structures were characterized by XRD (DX2700, China) at a scan rate (2θ) of 2° min^{-1}, operating at Cu Ka radiation (*l*=1.548 Å) with an accelerating voltage of 40 kV and an applied current of 30 mA. transmission electron microscopy (TEM), high-resolution TEM (HRTEM) and high-angle annular dark-field scanning transmission electron microscope (HAADF-STEM) with energy-dispersive X-ray spectrometry (EDS) images were obtained using a FEI Tecnai F20 electron microscope with an accelerating voltage of 200 kV. The morphology and structure of the samples were investigated by field emission scanning electron microscopy (FESEM) (JSM-7001F, JEOL, Tokyo, Japan). The specific surface area was calculated by using the Brunauer-Emmett-Teller (BET) method from data in a relative pressure (P/P_o) range between 0.05 and 0.20. Pore size distribution plots were derived from the adsorption branch of the isotherms based on the

Barrett-Joyner-Halenda (BJH) model. The chemical composition was investigated by X-ray photoelectron spectroscopy (XPS) using an ESCALab250 electron spectrometer (Thermo Scientific Corporation) with monochromatic 150 W Al Ka radiation. Raman spectra were recorded on a microscopic confocal Raman spectrometer. The instrument was fitted with 514.5 nm lasers. The amount of cobalt clusters was measured by thermos gravimetric analysis (TGA) (SDT Q600) from RT to 900 °C in air at a heating rate of 10 °C min^{-1}.

Electrochemical Measurements: The pretreatment of experiment was as follows: the glassy carbon electrode modified by the catalyst is used as the working electrode, Ag/AgCl as the reference electrode and a platinum wire as counter electrode. The working electrodes were polished alumina powder. And then 3.0 mg of each grinded catalyst were dispersed in the solvent mixture of (5 wt%) Nafion (30 µL), ethanol absolute (250 µL), and deionized water (250 µL) by sonication for 60 min, which were used as ORR working electrode. Afterwards, 15 µL of the catalyst ink was pipetted onto the polished glassy carbon electrode of 4 mm in diameter (loading ~ 673 µg cm^{-2}), and then glassy electrode was dried at room temperature in air before measurement.

ORR: ORR measurements were conducted using an electrochemical workstation (CHI 760E) with a typical three-electrode system. Cyclic voltammetry (CV) experiments with a sweep rate of 50 mV s^{-1} were recorded in the potential range of 0 to −1.0 V vs. Ag/AgCl. LSV in O_2-saturated 0.1 M KOH were recorded at a scan rate of 5 mV s^{-1} at various electrode rotation rates from 400 to 2500 rpm. All potentials reported in this work were converted from the Ag/AgCl to the RHE scale in 0.1 M KOH ($E_{RHE}= E_{Ag/AgCl} + 0.0591pH +0.197$). For the rotating ring-disk electrode (RRDE) measurements was conducted with the rotating speed fixed at 1600 rpm and the ring potential was set at 0.5 V with a scan rate of 10 mV s^{-1}. The electron transfers number (n) and the peroxide percentage (H_2O_2%) was calculated by the following equation:

$$\mathrm{n} = \frac{4NI_\mathrm{D}}{NI_D + I_R} \qquad (1)$$

$$\mathrm{H_2O_2(\%)} = \frac{200I_R}{NI_D + I_R} \qquad (2)$$

where I_R is the ring current, I_D is disk current and N = 0.43 is the current collection efficiency of the Pt ring.

Zinc-air battery test: To construct the ZAB, a zinc plate was used as an anode, and 6 M KOH was used for the electrolyte. The air electrode was prepared by uniformly coating dispersed solution of catalyst ink onto porous carbon paper (1 cm^{-2}) to achieve 1 mg cm^{-2} loading and then drying it at room temperature. An assembled ZAB was performed at different discharge currents.

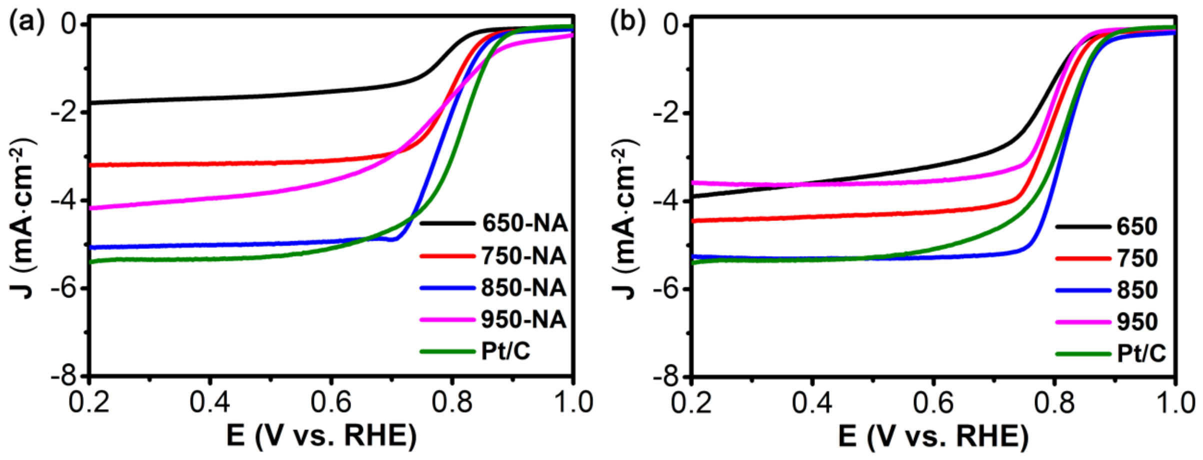

Figure S1. (a) LSV curves of GO (5 wt%) and Co-MOF at different carbonization temperature comparison with 20 wt% Pt/C at 1600 rpm (NA-not acid washing) and (b) acid washing.

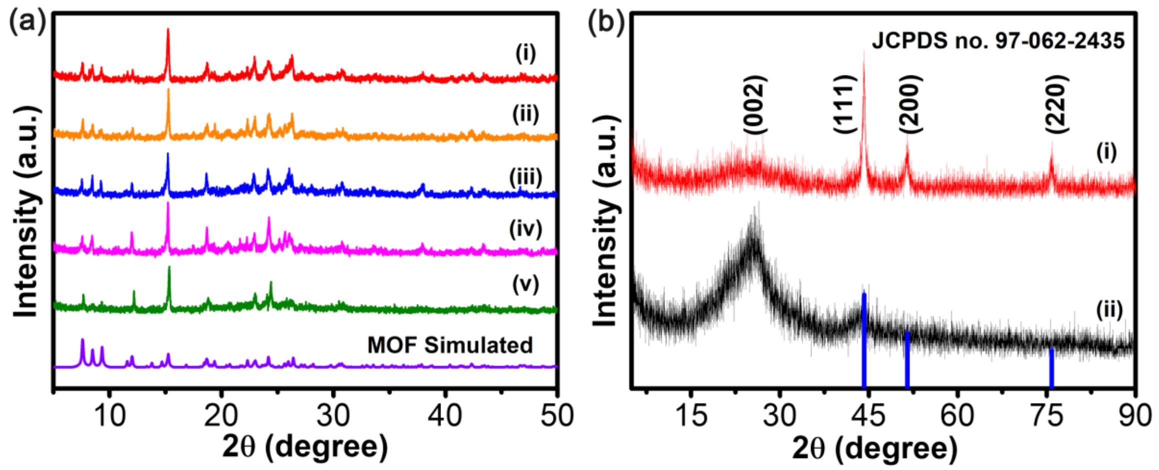

Figure S2. (a) XRD pattern of (i) Co-MOF; (ii) Co-MOF/GO (2 wt%); (iii) Co-MOF/GO (5 wt%); (iv) Co-MOF/GO (10 wt%); (v) Co-MOF/GO (15 wt%); (b) XRD pattern of (i) Co/N-r-GO-5%-850-NA; (ii) Co/N-r-GO-5%-850.

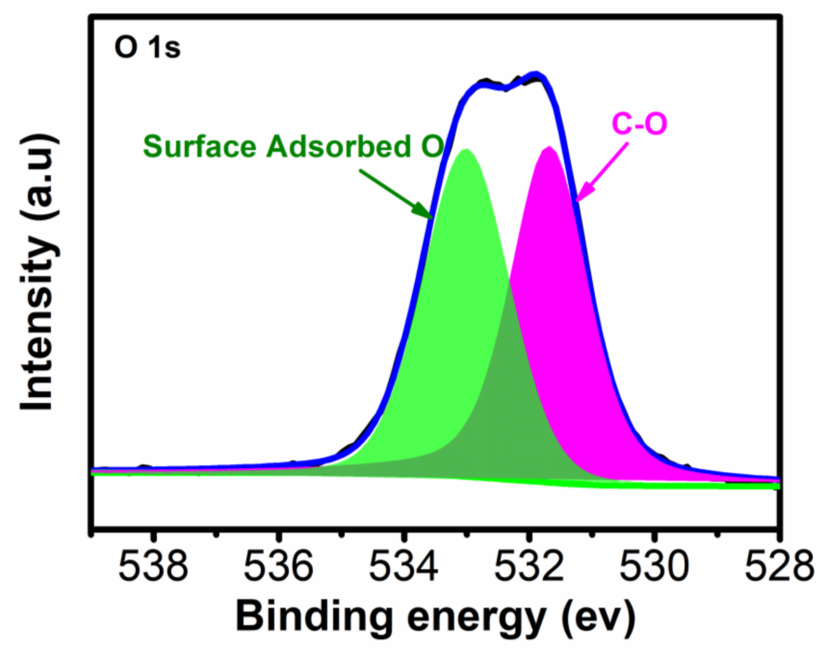

Figure S3. High resolution XPS for O 1s peak of Co/N-r-GO-5%-850.

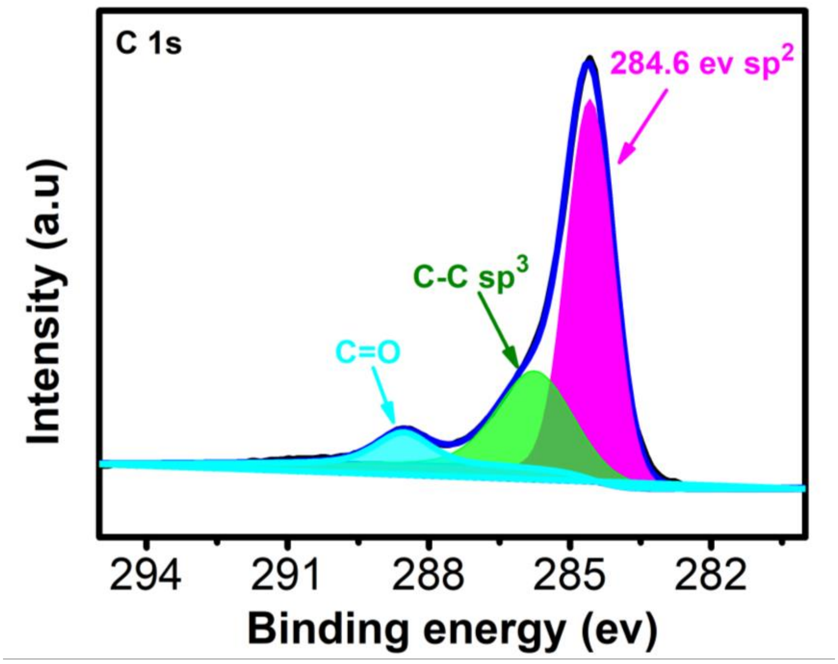

Figure S4. High resolution XPS for C1s peak of Co/N-r-GO-5%-850.

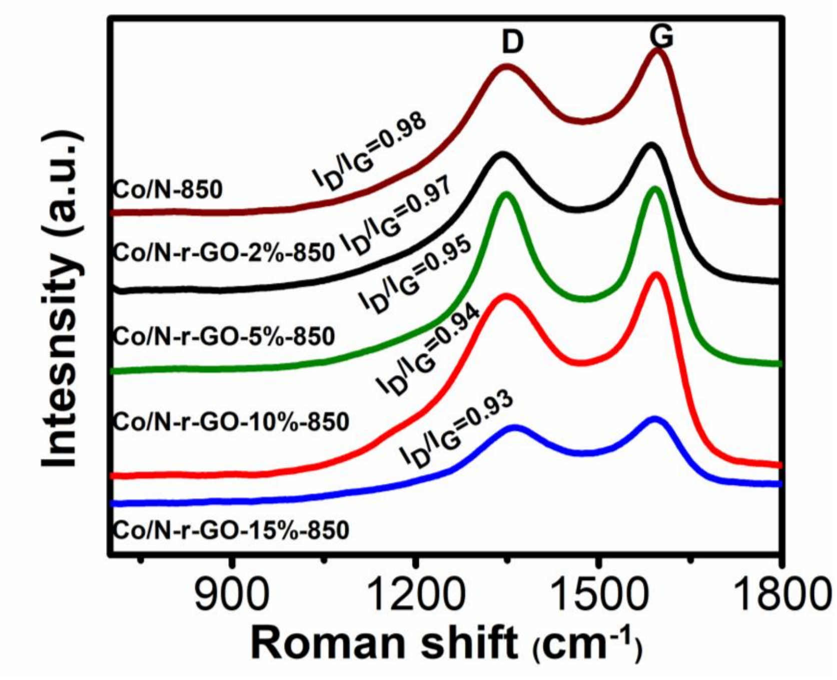

Figure S5. Raman spectra of Co/N-850, Co/N-r-GO (-2, -5, -10 and -15%)-850 materials.

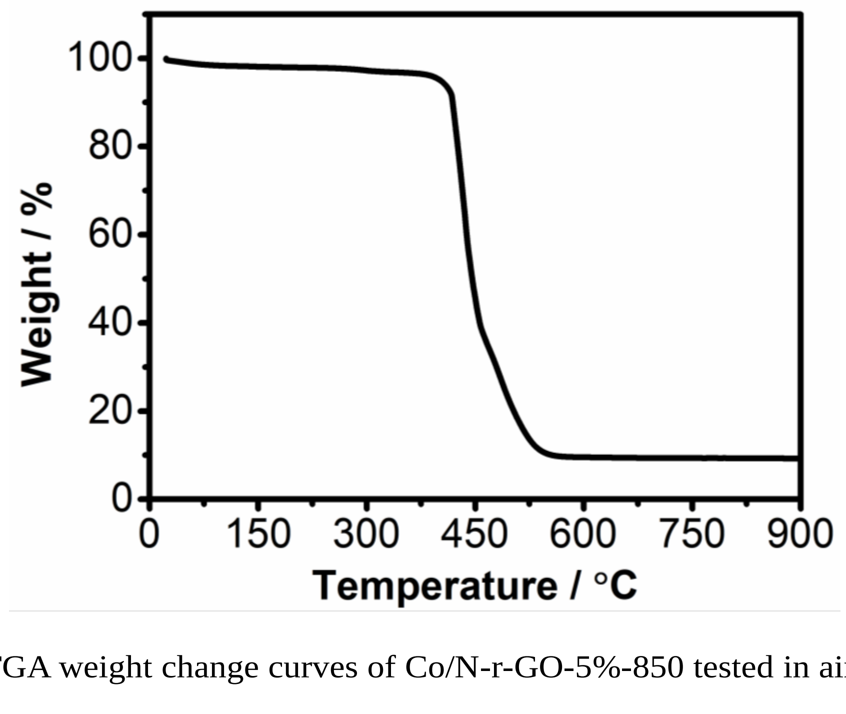

Figure S6. TGA weight change curves of Co/N-r-GO-5%-850 tested in air with a ramp rate of 10 ºC/min.

Table S1. The Brunauer-Emmett-Teller (BET) surface area, pore volume and average pore size of Co/N-r-GO (-2, -5, -10 and -15 %)-850 samples.

Sample	Co/N-r-GO-2 %-850	Co/N-r-GO-5 %-850	Co/N-r-GO-10 %-850	Co/N-r-GO-15 %-850
**Surfae area(m^2 g^{-1})**	165	179	198	206
**Pore Volume(cm^3 g^{-1})**	0.328	0.339	0.383	0.197
**Average pore size(nm)**	7.9	7.5	7.7	3.8

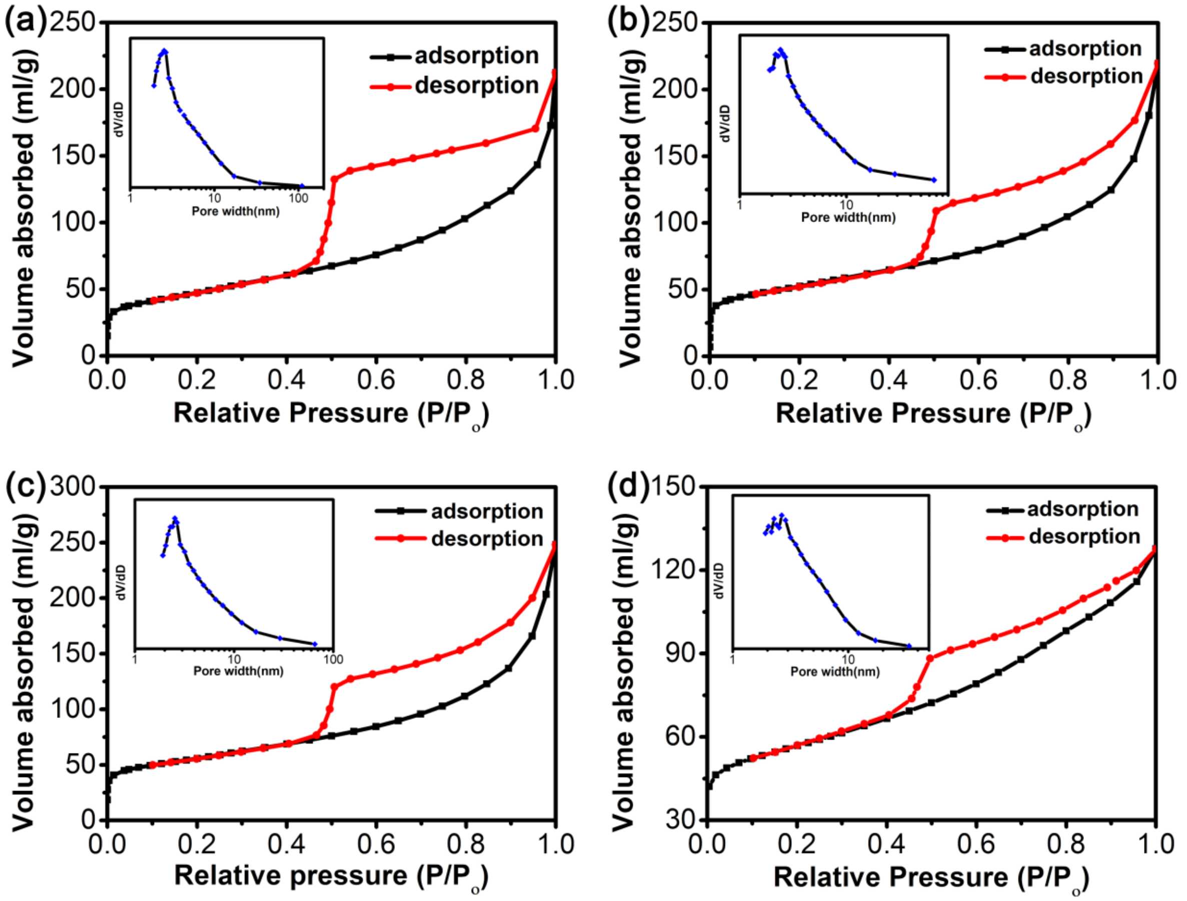

Figure S7. Nitrogen adsorption-desorption isotherms and inset pore size distributions (a) Co/N-r-GO-2%-850; (b) Co/N-r-GO-5%-850; (c) Co/N-r-GO-10%-850; (d) Co/N-r-GO-15%-850.

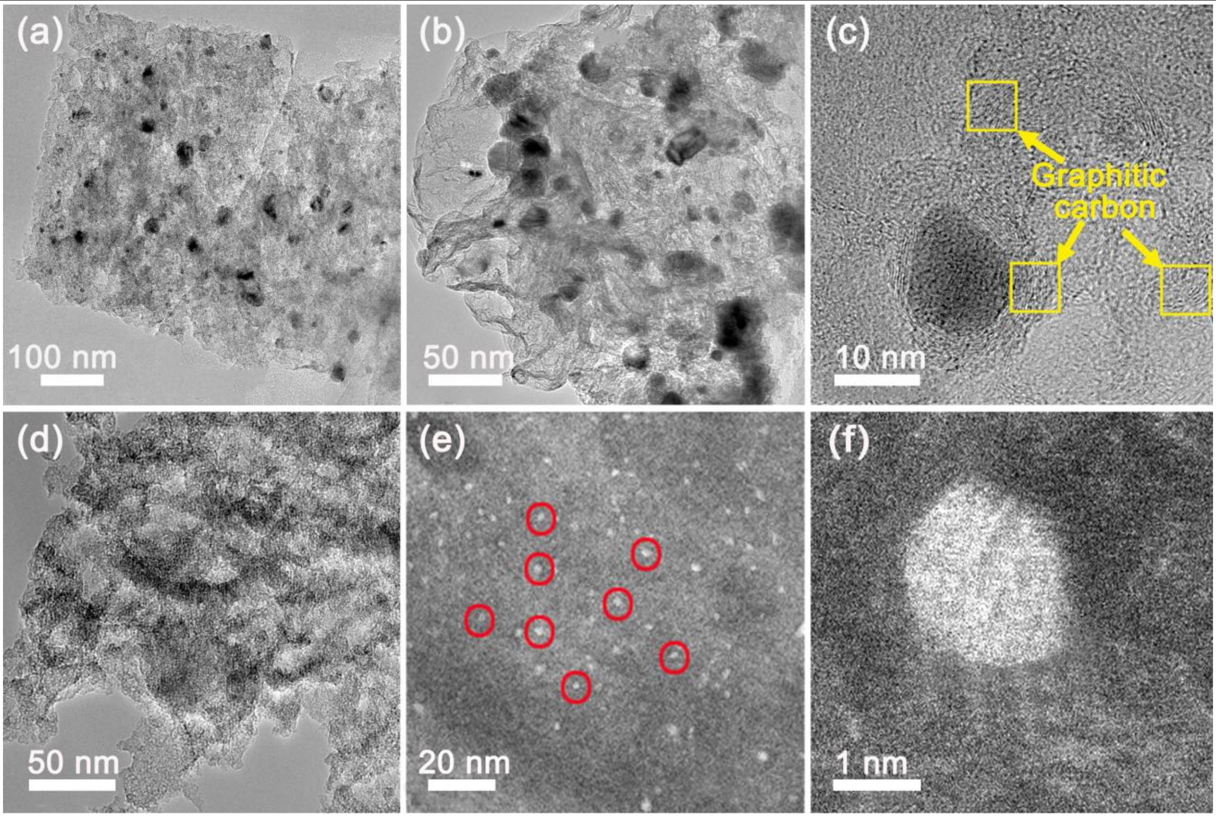

Figure S8. Co/N-r-GO-5%-850-NA images of (a-b) TEM and (c) HRTEM; Co/N-r-GO-5%-850 images of (d) TEM and (e-f) HAADF-STEM.

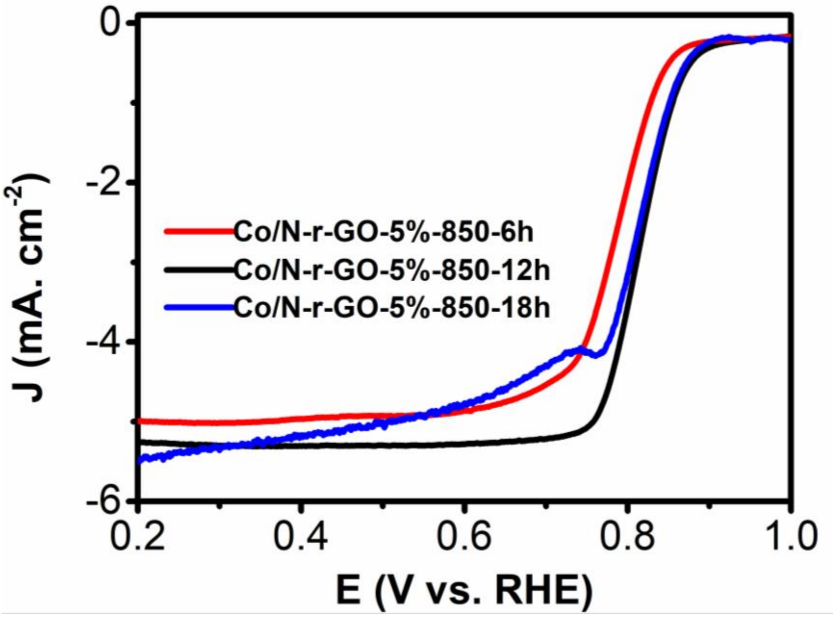

Figure S9. LSV curve of Co/N-r-GO-5%-850 comparison with different time at 1600 rpm.

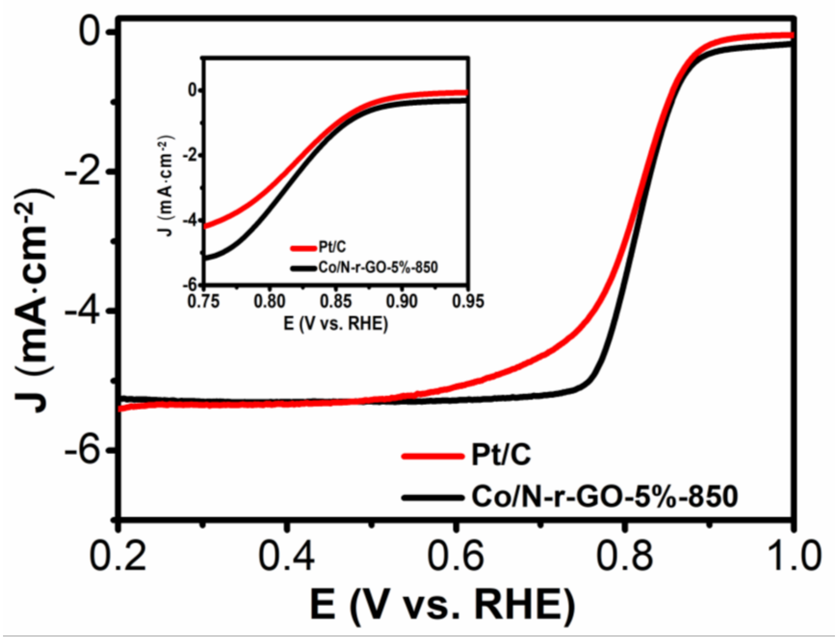

Figure S10. LSV curve of Co/N-r-GO-5%-850 comparison with Pt/C at 1600 rpm. The inset one shows the LSV curves from 0.75 V to 0.95 V.

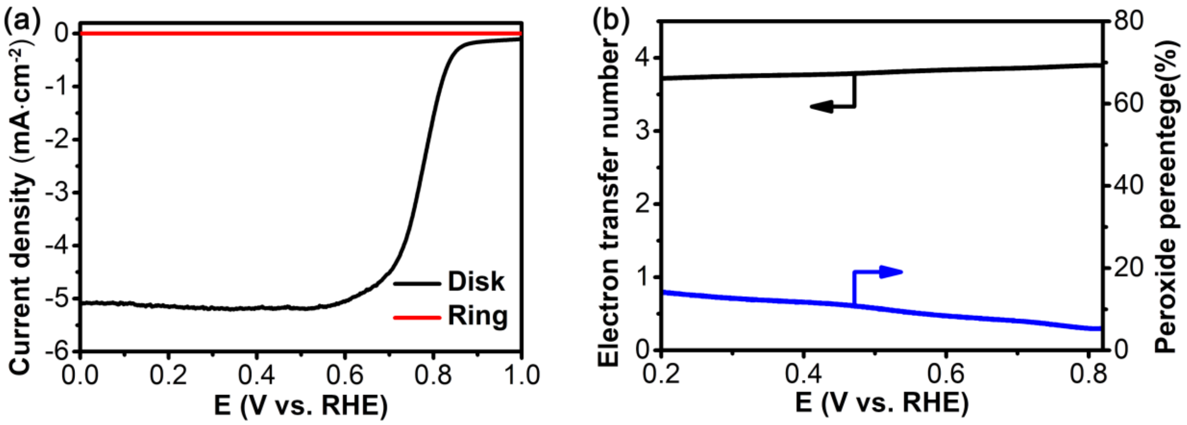

Figure S11. (a) Rotating ring-disk electrode (RRDE) voltammograms recorded with the Co/N-r-GO-5%-850 in O_2-saturated 0.1 M KOH at 1600 rpm; (b) The calculated percentage of peroxide and the electron transfer number (n).

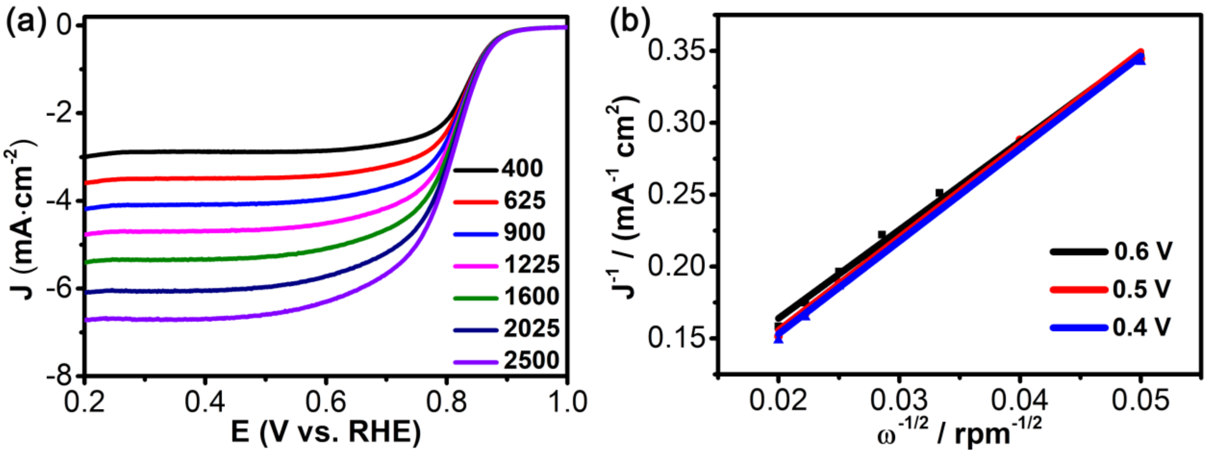

Figure S12. ORR performance of 20 wt% Pt/C (a) LSV curves at various rotation speeds; (b) the K-L plots from LSV curves at 0.6 V, 0.5 V, 0.4 V.

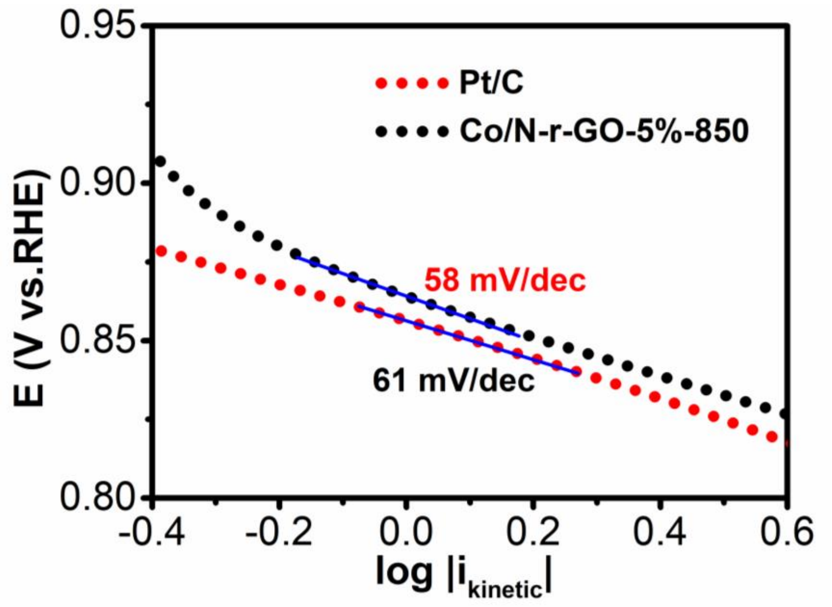

Figure S13. ORR Tafel plots of Co/N-r-GO-5%-850 with Pt/C in O_2-saturated 0.1 M KOH. The kinetic current density ($i_{kinetic}$) was calculated by the following equation:

$$i_{\text{kinetic}} = \frac{i_{\text{measured}} \times i_{\text{limited}}}{i_{\text{limited}} - i_{\text{measured}}}$$

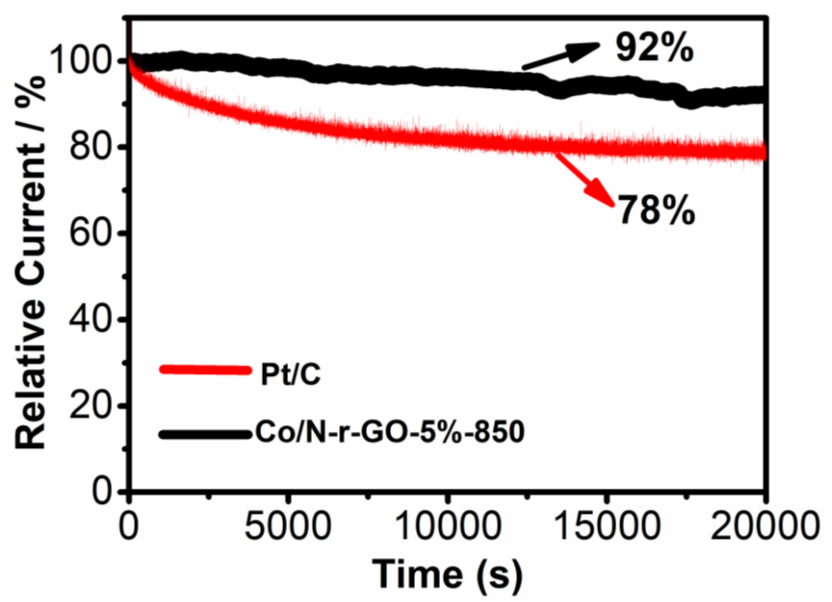

Figure S14. Long-term stability of Co/N-r-GO-5%-850 and Pt/C.

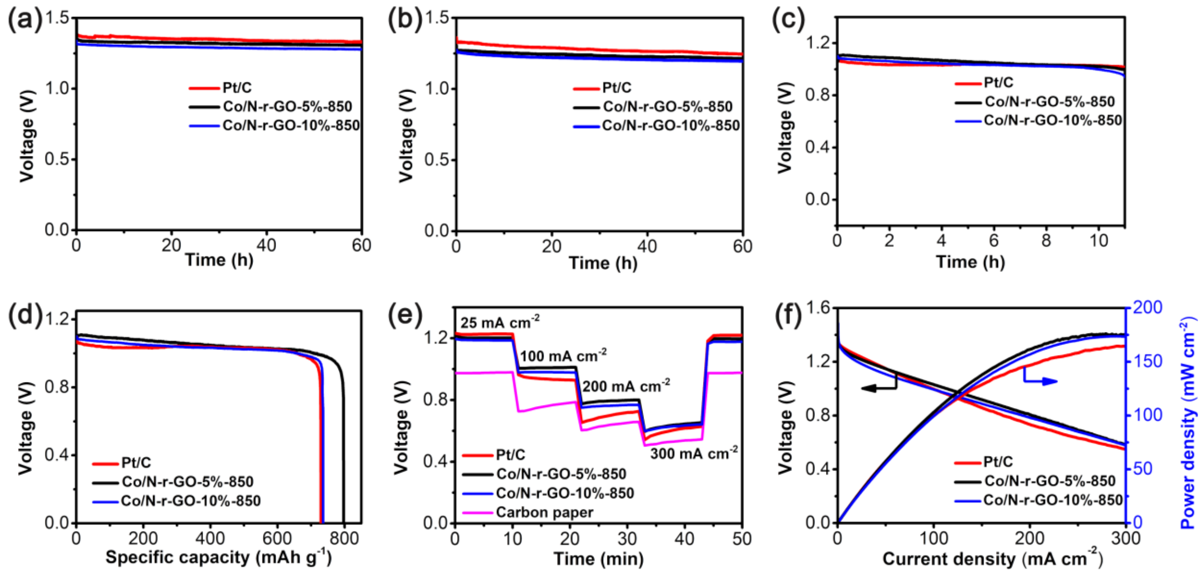

Figure 15. Curves of ZABs driven by Co/N-r-GO-5%-850, Co/N-r-GO-10%-850 and 20% Pt/C: typical galvanostatic discharge curves at current densities of (a)1 mA cm^{-2}; (b) 5 mA cm^{-2}; (c) and 50 mA cm^{-2}; (d) specific capacities normalized to the mass of the consumed zinc of ZABs at current density of 50 mA cm^{-2}; (e) the Zn-air batteries from low current densities to high current densities; (f) the polarization and power density curves.

References

[1] L. Schlechte, V. Bon, R. Grünker, N. Klein, I. Senkovska, S. Kaskel, *Polyhedron* **2012**, *44*, 179.

[2] Z. Xia, L. An, P. Chen, D. Xia, *Adv. Energy Mater*. **2016**, *6*, 1600458.

[3] S. Xu, P. Wu, *J. Mater. Chem. A*. **2014**, *2*, 13682.
